# Integrin *α*9 Suppresses Hepatocellular Carcinoma Metastasis by Rho GTPase Signaling

**DOI:** 10.1155/2018/4602570

**Published:** 2018-05-24

**Authors:** Yan-Li Zhang, Xin Xing, Li-Bo Cai, Lei Zhu, Xiao-Mei Yang, Ya-Hui Wang, Qin Yang, Hui-Zhen Nie, Zhi-Gang Zhang, Jun Li, Xue-Li Zhang

**Affiliations:** ^1^State Key Laboratory of Oncogenes and Related Genes, Shanghai Cancer Institute, Renji Hospital, Shanghai Jiao Tong University School of Medicine, Shanghai 200240, China; ^2^Department of Obstetrics and Gynecology, Fengxian Hospital, Shanghai 201499, China; ^3^Department of Accident and Emergency, Heihe No. 1 People's Hospital, Heihe 164300, China

## Abstract

Integrin subunit alpha 9 (ITGA9) mediates cell-cell and cell-matrix adhesion, cell migration, and invasion through binding different kinds of extracellular matrix (ECM) components. However, its potential role and underlying molecular mechanisms remain unclear in hepatocellular carcinoma (HCC). Here, we found that ITGA9 expression was obviously decreased in patients with HCC, which was negatively correlated with HCC growth and metastasis. ITGA9 overexpression significantly inhibited cell proliferation and migration *in vitro* as well as tumor growth and metastasis *in vivo*. Our data demonstrated that the inhibitory effect of ITGA9 on HCC cell motility was associated with reduced phosphorylation of focal adhesion kinase (FAK) and c-Src tyrosine kinase (Src), disrupted focal adhesion reorganization, and decreased Rac1 and RhoA activity. Our data suggest ITGA9, as a suppressor of HCC, prevents tumor cell migration and invasiveness through FAK/Src-Rac1/RhoA signaling.

## 1. Introduction

Hepatocellular carcinoma (HCC) is a highly malignant solid tumor which results in chronic inflammation in the liver [1]. Until now, there is no effective drug for the treatment with advanced HCC patients [2]. The principal character of HCC is early metastasis and poor prognosis. A series of changes in the tumor microenvironment (TME) are involved in the progression of HCC [3]. The adaptation of cancer cells to its surrounding microenvironment depends on the interaction between the extracellular matrix (ECM) with membrane receptors [4]. Many molecules in TME have been reported to influence tumor development by regulating tumor cell proliferation, apoptosis, and motility [5–7]. It has been shown that integrin receptors and its downstream signal molecules, including Src, FAK, and p130Cas, have a remarkable influence on tumor progression and metastasis [8].

Integrins are heterodimeric integral membrane glycoproteins composed of noncovalently associated *α*- and *β*-subunits forming 24 heterodimers that recognize distinct but overlapping ligands, which can mediate cell adhesion, migration, and proliferation [9–11]. Different integrins are involved in different cellular processes, such as cell attachment to ECM, cell proliferation, and cell motility, which can be used as therapeutic targets in cancer [12].

Integrin *α*9 subunit, which pairs only with integrin *β*1 subunit, mediates the binding to a large number of ECM components to affect cell adhesion and motility. There is a key role of integrin *α*9*β*1 in lymphangiogenesis and angiogenesis [13, 14]. Integrin *α*9*β*1 is expressed not only by several human normal cells but also by different kinds of human cancer cells and closely correlated with tumor grade [15, 16]. It has been reported that integrin *α*9*β*1 in colon carcinoma is linked to tumor cell proliferation and migration by enhanced epithelial-mesenchymal transition (EMT) [17].

However, the biological functions of ITGA9 in HCC and the underlying molecular mechanisms have not been studied yet, therefore placing the restrictions on developing novel anticancer-targeted therapies. In this study, we investigated the role of ITGA9 in HCC and the underlying mechanisms involved in its function, trying to provide a new potential target for HCC treatment.

## 2. Materials and Methods

### 2.1. Cell Cultures

Normal human liver cell line THLE-2 was from American Type Culture Collection (ATCC). HCC cell lines HuH7, Hep3B, HepG2, SMMC-7721, MHCC-LM3, MHCC-97 L, and MHCC-97H have been described previously [4, 18]. All of these cells were cultured in a specific medium according to ATCC instructions, supplemented with 10% heat-inactivated fetal bovine serum (FBS) and 1% penicillin/streptomycin, and incubated in a humidified incubator under 5% CO_2_ at 37°C.

### 2.2. Clinical Samples

HCC tissue microarrays containing 202 HCC samples, 131 pairs of primary HCC, and their corresponding noncancerous liver (CNL) tissues were obtained from HCC patients treated at the Department of Transplantation and Hepatic Surgery, Renji Hospital (RJH). All human specimens were received from patients who underwent surgical resection and signed informed consent before their operations. The research was approved by the Research Ethics Committee of Ren Ji Hospital, Shanghai Jiao Tong University School of Medicine.

### 2.3. Immunohistochemical and H&E Staining

Immunohistochemical staining and H&E staining were performed as described previously [19]. Anti-ITGA9 (ab140599; Abcam) antibody was used. To quantify the level of ITGA9 protein expression, each tumor was assigned with a score according to the intensity of cell membrane staining and the proportion of stained tumor cells (score 0 = 0–5%, score 1 = 6–30%, score 2 = 31–70%, and score 3 = 71–100%). Two pathologists quantified ITGA9 protein level independently in a blinded manner (low expression group: score 0-1, high expression group: score 2-3).

### 2.4. Lentivirus Production and Cell Transduction

The human ITGA9 ORF (NM_002207.2) was subcloned into the pEZ-lv105 vector (GeneCopoeia, China) to generate pEZ-lv105-ITGA9 plasmid. Virus packaging and cell transduction were performed as previously reported [19].

### 2.5. Cell Apoptosis Assay

Apoptotic cells were analyzed by annexin V-FITC staining and PI labeling as previously described [20]. SMMC-7721 and MHCC-LM3 cells with Lenti-vector or Lenti-ITGA9 were cultured and used in this assay.

### 2.6. Quantitative Real-Time PCR (qPCR)

Total RNA from HCC cell lines was extracted by using TRIzol (Invitrogen, Carlsbad, CA, USA). Reverse transcription was performed as previously described [21]. *β*-Actin was used as internal control for quantification. The data were analyzed using the 2^−ΔΔCt^ approach. For ITGA9 mRNA level in HCC cell lines, ΔCt value of THLE-2 cell line was used as the reference. Primer sequences used in our study were as follows: ITGA9-F, 5′-CCCAGAAGAGGTGACGG-3′; ITGA9-R, 5′-GCAGCAGGAAGATG AGGA-3′; *β*-actin-F, 5′-TGTGGGGCGCCCCAGGCACCA-3′; and *β*-actin-R, 5′-CTCC TTAATGTCACGCACGATTTC-3′.

### 2.7. Western Blot

Western blots were performed as described previously [7]. The primary antibodies against ITGA9 (ab140599; Abcam), FAK (ab81293; Abcam), p-FAK Tyr397 (ab81298; Abcam), Src (2108; Cell Signaling Technology), p-Src Tyr527 (2105; Cell Signaling Technology), and *α*-tubulin (T6199; Sigma-Aldrich) were incubated for overnight at 4°C, followed by incubating with species-specific antibodies (926–32213; 926–68051; LI-COR, Lincoln, NE) for 1 h. The signals were detected by Odyssey infrared imaging system (LI-COR, Lincoln, NE) and further quantified by ImageJ software.

### 2.8. Cell Viability and Colony Formation Assay

The cell viability and colony formation were determined as previously described [22]. To determine cell viability, 2000 cells/well were seeded into a 96-well plate and detected by Cell Counting Kit-8 (CCK8, Dojindo, Japan) after 0, 1, 2, 3, and 4 days, respectively. For flat plate clone formation, 1000 cells/well were seeded into a 6-well plate and grown for 14 days followed by staining with 0.1% crystal violet solution in 20% methanol. The experiments were done in triplicate and repeated twice.

### 2.9. Transwell Assay

The ability of cell motility was detected by using transwells with 12 *μ*m pores (Merck Millipore) as previously described [23]. 2 × 10^4^ HCC cells in 200 *μ*l culture medium without FBS were seeded on the upper chamber, and 600 *μ*l medium with 5% FBS was injected into the lower chamber. For invasion assay, 100 *μ*l Matrigel (BD Bioscience, USA) was placed on the upper chamber. Cell numbers were scored from six random areas of each well.

### 2.10. Xenograft Studies

Xenograft studies were performed as previously described [4]. All mice were sacrificed after 6 weeks, and the xenograft was stripped out and weighed for further analysis.

### 2.11. Immunofluorescence Staining

Cells were seeded at 12-well U-Chamber (Ibidi, Germany), fixed with 4% paraformaldehyde for 15 min, and permeabilized with 0.05% Triton X-100 for 1 min at room temperature. Primary antibodies used in immunofluorescence staining were vinculin (EPR8185; Epitomics) and paxillin (ab32084; Abcam). The nucleus was stained with DAPI (Sigma-Aldrich, USA). Images were acquired by confocal microscopy (LSM 510, METALaser Scanning Microscope, Zeiss). The raw density was assessed using ImageJ.

### 2.12. Pull-Down Assay

Cells were serum-starved overnight and stimulated with LPA. Active small G-proteins were detected by pull-down with GST-RBD and GST-CRIB. Anti-RhoA (2117; Cell Signaling Technology), anti-Rac1 (2320346; Merck Millipore), and anti-Cdc42 (2466; Cell Signaling Technology) against the corresponding small G-protein were used for immunoblotting in this assay. The active and total GTPases were subsequently detected with horseradish peroxidase- (HRP-) conjugated secondary antibody according to the manufacturer's recommendations.

### 2.13. Statistical Analysis

Statistical analysis was performed using Student's *t*-test for two groups or ANOVA for multiple groups. All quantitative data presented are mean ± standard error of mean (SEM) of at least three experiments. *P* < 0.05 was considered statistically significant. Graphical representation was created by GraphPad Prism 5 software (San Diego, USA).

## 3. Results

### 3.1. ITGA9 Is Significantly Downregulated in HCC and Correlates with Vascular Invasion and Prognosis

**Figure 1:**
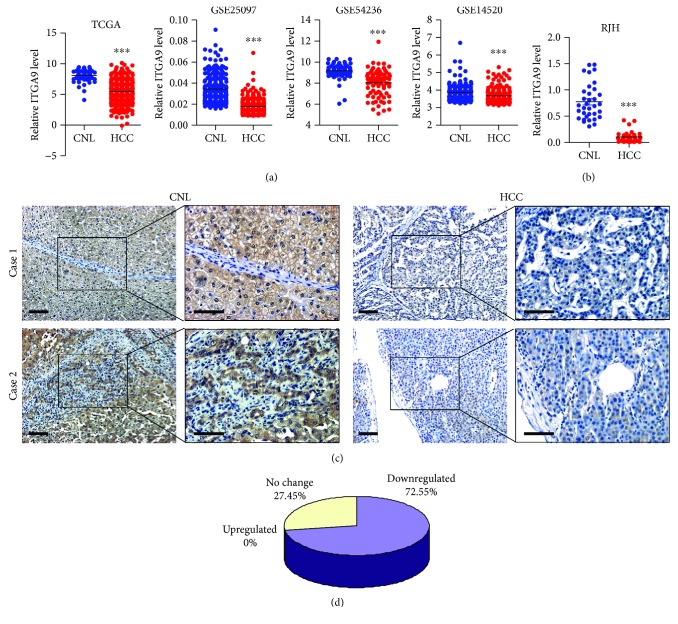
Analysis of ITGA9 expression in HCC tissues. (a) Analysis of ITGA9 expression in HCC mRNAseq data from the TCGA database (*n* = 372) and 3 independent HCC microarray datasets from the GEO database (GSE25097, *n* = 268; GSE54236, *n* = 161; and GSE14520, *n* = 225). Values are means ± SEM. ^∗∗∗^*P* < 0.001. (b) Expression levels of ITGA9 in CNL tissues and HCC tissues by qPCR for 34 pairs of the CNL/HCC tissues from RJH. Values are means ± SEM. ^∗∗∗^*P* < 0.001. (c) Representative images of ITGA9 immunohistochemical staining in 131 paired HCC and CNL tissues. Scale bars, 100 *μ*m. (d) Graphical analyses of (c) showing the decreased ITGA9 level in HCC patients compared to the paired CNL. Score of CNL > HCC: 72.55% (*n* = 95), score of CNL = HCC: 27.45% (*n* = 36), and score of CNL < HCC: 0% (*n* = 0).

To explore biological functions of ITGA9 in HCC, we first analyzed ITGA9 expression using the TCGA and the GEO databases. We found that ITGA9 mRNA level was downregulated in HCC compared to CNL tissues (Figure 1(a)). For validation, we next investigated the expression of ITGA9 in HCC tissue microarray by qPCR and immunohistochemical staining. Consistently, HCC tissues showed significantly decreased ITGA9 expression compared to normal-matched tissues (Figures 1(b) and 1(c)). Statistical analysis showed the decreased ITGA9 level in 72.55% of HCC patients compared to the paired CNL (Figure 1(d)).

Furthermore, ITGA9 protein level associated well with alpha-fetoprotein, vascular invasion, tumor thrombosis, tumor size, and TNM stage (Table 1). Similar results were also obtained from GSE14520 microarray datasets. ITGA9 mRNA and protein levels were closely correlated with ALT, TNM staging, BCLC staging, and CLIP staging in the HCC tissues (Table 2).

### 3.2. ITGA9 Affects HCC Cell Growth Both *In Vitro* and *In Vivo*

**Figure 2:**
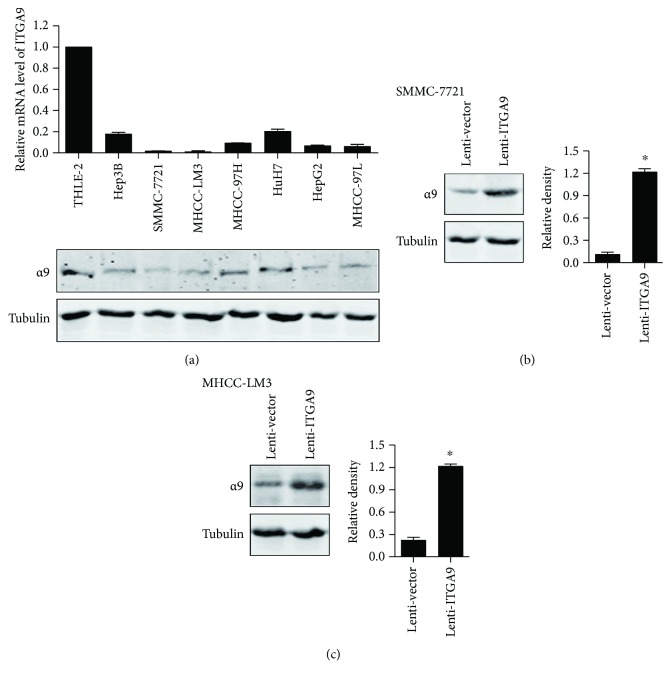
Analysis of ITGA9 expression in cell lines. (a) ITGA9 levels in 7 HCC cell lines and the immortalized human liver cell line THLE-2 as measured by qPCR and Western blot. (b and c) Expression of ITGA9 in SMMC-7721 and MHCC-LM3 cells with Lenti-vector or Lenti-ITGA9. Tubulin was used as an internal control. Values are means ± SEM. ^∗^*P* < 0.05.

**Figure 3:**
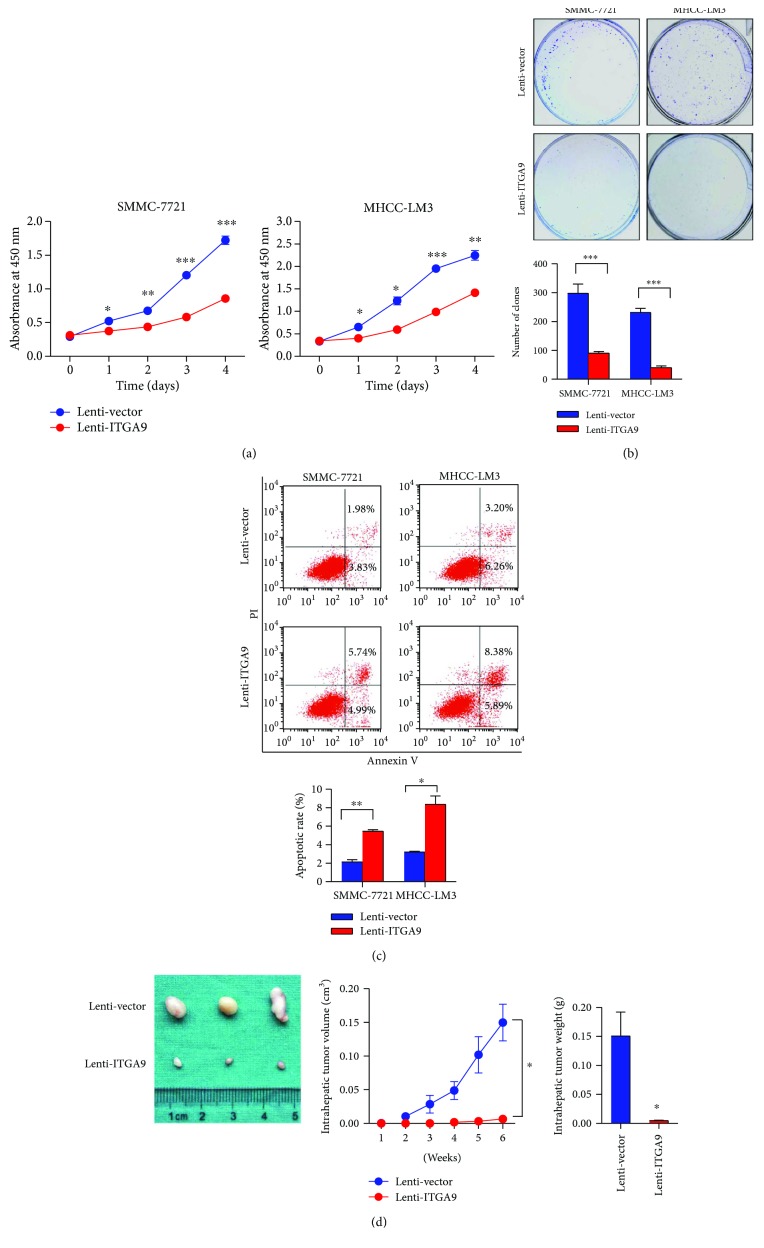
ITGA9 prevents HCC growth *in vitro* and *in vivo*. (a) Analysis of HCC cell viability with ITGA9 overexpression or control by CCK8. *n* = 6. (b) Analysis of HCC cell proliferation with ITGA9 overexpression or control by colony formation. (c) Annexin V/PI staining was used to measure apoptosis in HCC cells. Numbers indicated the percentage of each quadrant. *n* = 3. (d) *In vivo* orthotopic growth of ITGA9-overexpressed versus control HCC cells. *n* = 6. Values are means ± SEM. ^∗^*P* < 0.05, ^∗∗^*P* < 0.01, and ^∗∗∗^*P* < 0.001.

To elucidate the relevance of ITGA9 and HCC progression, we first analyzed ITGA9 level in HCC cell lines. Compared with immortalized normal liver cell THLE-2, ITGA9 mRNA and protein levels were expressed lower in most of the examined HCC cell lines (Figure 2(a)). Then, we stably overexpressed ITGA9 expression in SMMC-7721 and MHCC-LM3 cells (Figures 2(b) and 2(c)). In CCK8 assay, ITGA9 overexpressing significantly decreased viability of HCC cells (Figure 3(a)). In plate clone formation assay, ITGA9 overexpression dramatically reduced colony formation of the HCC cells, manifested in the number of clones (Figure 3(b)). Moreover, cell apoptosis was obviously increased in both ITGA9-overexpressing SMMC-7721 and MHCC-LM3 cells (Figure 3(c)). Furthermore, the effect of ITGA9 overexpressing on tumorigenesis was evaluated in xenografts *in vivo*. The volume and weight of the tumors from Lenti-ITGA9 cells were clearly attenuated compared to the tumors from control cells (Figure 3(d)). Taken together, these results show an inhibitory function of ITGA9 in HCC growth.

### 3.3. ITGA9 Inhibits HCC Cell Metastasis Both *In Vitro* and *In Vivo*

**Figure 4:**
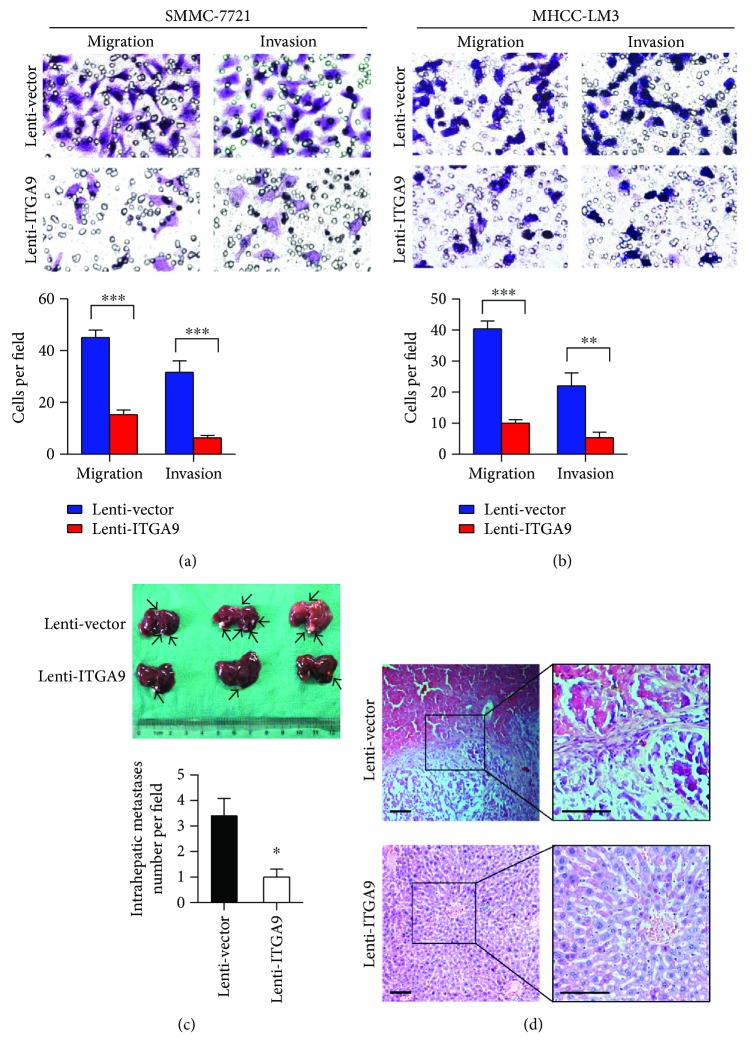
ITGA9 suppresses HCC cell motility *in vitro* and *in vivo*. (a and b) Transwell migration and invasion assays in ITGA9-overexpressed or control HCC cells. Quantification of 6 randomly selected fields in each assay. Original magnification: 200x. (c) *In vivo* orthotopic metastases of ITGA9-overexpressed versus control HCC cells. Black arrows indicate metastases. (d) H&E staining of the mouse liver tissues. Scale bars, 100 *μ*m. Data are obtained from three representative experiments. Values are means ± SEM. ^∗^*P* < 0.05, ^∗∗^*P* < 0.01, and ^∗∗∗^*P* < 0.001.

Then, we examined ITGA9 functions in cell migration and invasiveness *in vitro*. HCC cells with ITGA9 overexpression migrated obviously slower, and their invasion efficiency significantly decreased compared with control cells (Figures 4(a) and 4(b)). Additionally, ITGA9 overexpression or control cells were orthotopically injected into nude mice to evaluate the ability of metastases *in vivo*. Metastatic modules from Lenti-ITGA9 cells displayed less than those from Lenti-vector cells (Figure 4(c)). And histological staining indicated that intrahepatic metastasis was strongly inhibited by ITGA9 overexpression in HCC cells (Figure 4(d)). These results demonstrate that ITGA9 plays a crucial role in HCC cell invasiveness and metastasis.

### 3.4. ITGA9 Overexpression Disrupts Focal Adhesion Assembly, Inactivates Rac1/RhoA, and Reduces FAK/Src Phosphorylation

**Figure 5:**
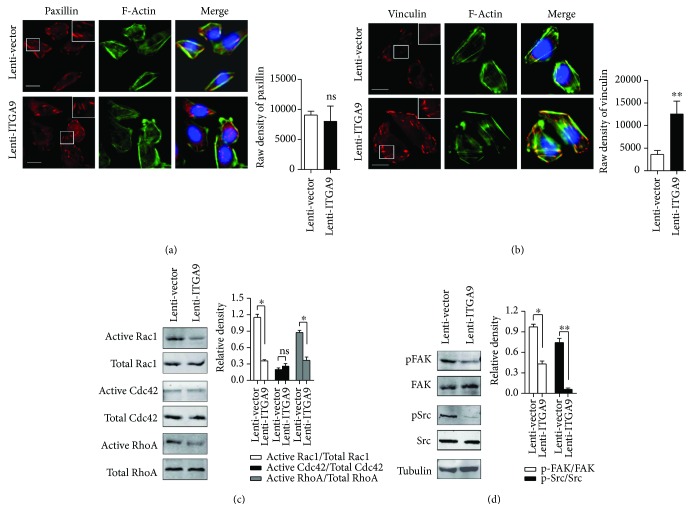
ITGA9 affects focal adhesions and Rho GTPase activity. (a and b) Immunofluorescence staining of paxillin (red) and vinculin (red). F-Actin was stained by FITC phalloidin (green), and the cell nuclei were stained by DAPI (blue). Scale bars, 10 *μ*m. (c) Pull-down assays of the activities of RhoA, Cdc42, and Rac1 in ITGA9-overexpressed or control HCC cells. (d) FAK and Src phosphorylation of ITGA9-overexpressed versus control HCC cells. Tubulin was used as an internal control. Values are means ± SEM. ns: no significance. ^∗^*P* < 0.05 and ^∗∗^*P* < 0.01.

To uncover the underlying mechanisms of integrin *α*9-mediated suppression of HCC progression, we firstly explored the related pathway by analyzing the TCGA database. ITGA9 expression was closely associated with the pathways involved in cancer and regulation of actin cytoskeleton and focal adhesion, which was shown by KEGG pathway analysis (Table 3). It has been reported that integrin-mediated focal adhesion plays a curial role in controlling cell motility [24]. Since paxillin and vinculin represent newly formed and mature focal adhesion, respectively, we investigated these two adapter proteins in ITGA9-overexpressed and control cells. Compared with the control cells, ITGA9 overexpression cells showed no obvious difference in paxillin-positive adhesion plaque, whereas displaying more vinculin-positive adhesion plaque (Figures 5(a) and 5(b)).

It is well known that cytoskeleton rearrangement and focal adhesion formation are orchestrated by small G-proteins, which play key roles in the motility of cancer cells. By pull-down assay, we found the activity of Rac1 and RhoA decreased significantly in ITGA9 overexpression cells. However, there was no significant difference detected in Cdc42 activity between ITGA9 overexpression and control cells (Figure 5(c)). The mechanism for ITGA9-mediated dysregulation of focal adhesion could also be related to FAK and Src, which are key adaptor molecules in adhesions. Indeed, the phosphorylation levels of FAK and Src were decreased in ITGA9 overexpression HCC cells compared to control cells (Figure 5(d)).

Taken together, ITGA9 overexpression-induced alterations, including increased vinculin-containing focal adhesions, decreased activity of Rac1 and RhoA, and reduced phosphorylation of FAK and Src, were conducive to the suppressive effects of ITGA9 on HCC cell behavior.

## 4. Discussion

Given that no dominant mechanism is responsible for HCC cell growth and metastasis, efforts aiming at identifying novel molecules may exert therapeutic benefits for patients suffering from HCC. Integrin receptors and associated signaling have shown to play important roles during HCC progression [25, 26]. In our current study, we demonstrated that ITGA9 expression was obviously downregulated in HCC patients. Our study is the first one to reveal that ITGA9 negatively correlated with HCC progression.

It has been reported that ITGA9 plays supportive roles in breast and small-cell lung cancer [27, 28]. Gupta et al. have shown that integrin *α*9*β*1 facilitates colon carcinoma growth and metastasis by enhancing EMT [17]. The high level of integrin *α*9*β*1 is positively related to the grade of glioblastoma [29]. However, ITGA9 has been previously reported to be downregulated in human squamous cell carcinoma of the head and neck [30], non-small-cell lung cancer [31], and oral squamous cell carcinoma [32]. Besides, ITGA9 mRNA level was significantly downregulated in bladder cancer tissues compared with the corresponding adjacent to the tumor tissues [33]. Nawaz et al. have reported that ITGA9 promoter was downregulated by aberrant hypermethylation in promoter and probably facilitated the process of nasopharyngeal carcinoma [34]. Nevertheless, our data revealed that ITGA9 not only restrains tumor growth but also suppresses tumor metastasis to prevent HCC progression. These results indicate that both ITGA9 expression level and its function are dependent on different cancer types, showing tumor heterogeneity.

Studies have noted that FAK and Src promote cancer cell motility by controlling the formation and turnover of focal adhesion through multiple signal pathways [8]. It has been reported that overactive small Rho GTPases play supportive roles in tumor progression. Rac-1 mutations can drive the malignancy of melanoma [35]. Gain-of-function mutations of RhoA occur specifically in poorly differentiated adenocarcinomas [36]. In this study, we demonstrated that ITGA9 reduced the FAK and Src phosphorylation, decreased Rac1 and RhoA activation, and promoted focal adhesion maturation, leading to the suppression of HCC cell motility.

In summary, our results first demonstrated that ITGA9 suppresses HCC cell migration and invasion via FAK/Src-Rho GTPase signaling. Furthermore, our findings indicated ITGA9 might be identified as a diagnostic biomarker for HCC and provided a potential solution for the treatment of HCC.

## Figures and Tables

**Table 1:** Correlation of clinicopathological features with ITGA9 expression.

Variable		ITGA9 (*n*)	
	High	Low	*P* value
Age			
≤50 years	63	40	0.081
>50 years	72	27	
Gender			
Female	14	14	0.042
Male	121	53	
^∗^Alpha-fetoprotein			
≤20 ng/ml	53	16	**0.025**
>20 ng/ml	80	51	
Gamma-glutamyltransferase			
≤50 (U/l)	48	27	0.536
>50 (U/l)	86	40	
Liver cirrhosis			
Yes	116	60	0.649
No	19	7	
Tumor multiplicity			
Single	109	59	0.191
Multiple	26	8	
Tumor satellite			
Yes	42	19	0.688
No	93	48	
Tumor encapsulation			
Incomplete	87	50	0.186
Complete	46	17	
Tumor thrombosis			**0.007**
Yes	22	22	
No	113	45	
Tumor differentiation			
I	3	0	0.056
II	55	17	
III	76	50	
^∗^Vascular invasion			
Yes	32	25	**0.043**
No	103	42	
^∗^Tumor size			
≤5 cm	76	25	**0.011**
>5 cm	59	42	
^∗^TNM stage			
I	83	35	**0.027**
I	17	6	
III	34	25	

^∗^ *P* < 0.05 (*n* = 202; Pearson's *χ*^2^ test).

**Table 2:** Correlation of clinicopathological features with ITGA9 expression in GSE14520 microarray data.

Variable		ITGA9 (*n*)	
	High	Low	*P* value
Age			
≤50 years	51	74	0.180
>50 years	38	79	
Gender			
Female	12	19	0.124
Male	77	134	
HBV status			
AVR-CC	19	39	0.164
CC	57	103	
N	2	4	
^∗^ALT			
>50 (U/l)	28	72	**0.017**
≤50 (U/l)	61	81	
AFP			
>300 ng/ml	49	61	0.052
≤300 ng/ml	38	90	
Main tumor size			
>5 cm	35	53	0.307
≤5 cm	53	100	
Multinodular			
No	67	123	0.351
Yes	22	30	
Cirrhosis			
No	7	12	0.995
Yes	82	141	
^∗^TNM staging			
0	11	6	**0.043**
I	31	65	
II	25	53	
III	89	29	
^∗^BCLC staging			
0	5	15	**0.035**
A	49	103	
B	12	12	
C	12	17	
^∗^CLIP staging			
0	26	72	**0.016**
1	28	51	
2	19	16	
3	4	5	
4	1	2	
5	0	1	
Predicted risk metastasis signature			
High	47	74	0.505
Low	42	79	
CGH_survival_group			
G1	11	8	0.063
G2	19	25	

^∗^ *P* < 0.05 (*n* = 242; Pearson's *χ*^2^ test).

**Table 3:** Gene set enrichment analysis (GSEA) of ITGA9 mRNA profiling results in HCC from the TCGA database.

Pathway	Genes (*n*)	*P* value	*Q* value
Pathways in cancer	314	0.0000	0.0627
Regulation of actin cytoskeleton	196	0.0000	0.0678
Focal adhesion	193	0.0000	0.0679
Purine metabolism	153	0.0000	0.0953
Cell adhesion molecules cams	128	0.0000	0.0574
Lysosome	121	0.0000	0.0752
Pyrimidine metabolism	97	0.0000	0.0645
ECM receptor interaction	83	0.0000	0.0758
Arrhythmogenic right ventricular cardiomyopathy	68	0.0000	0.0576
Non-small-cell lung cancer	53	0.0000	0.0687
Vasopressin-regulated water reabsorption	41	0.0000	0.0610
Prostate cancer	87	0.0019	0.0712
Small-cell lung cancer	84	0.0020	0.0639
PPAR signaling pathway	65	0.0020	0.0636
Chemokine signaling pathway	181	0.0020	0.0701
Leukocyte transendothelial migration	107	0.0020	0.0583
Basal cell carcinoma	54	0.0020	0.0657
Valine leucine and isoleucine degradation	44	0.0038	0.0632
Hedgehog signaling pathway	54	0.0039	0.0602
Inositol phosphate metabolism	54	0.0040	0.0664

## Data Availability

The data used to support the findings of this study are included within the article.

## Abbreviations

ITGA9: Integrin *α*9
HCC: Hepatocellular carcinoma
EMT: Epithelial-mesenchymal transition
CNL: Corresponding noncancerous liver
FAK: Focal adhesion kinase
Src: c-Src tyrosine kinase.
